# Academy of Sciences

**Published:** 1847-10

**Authors:** 


ACADEMY OF SCIENCES.

*Composition of the Blood in Scurvy.*—M. Marshal de Calvi, in an essay upon this subject, acknowledged the truth of Professor Andral's observation, that the quantity of fibrin might not be diminished in the blood of scorbutic patients, but accounted for the fact by the inflammatory condition generated in the circulating fluid by the efforts necessary for the resorption of extravasated blood. The singular coincidents of the diminution of albumen and of globulin, with the absence of dropsical effusion or of murmurs in the heart and arteries, M. Marshall explained by the difference which muse exist between diminution of the power of creating albumen in the blood, and genuine decrease or destruction of the component principle.

*Treatment of Cancer.*—M. Rivaillé read a paper on the use of caustics for the treatment of cancer. In a general manner Dr. Rivaillé stated that caustics were preferable to the removal of these tumours with the knife. Concentrated nitric acid had yielded him in his practice most advantageous results. Poured over a pledget of lint, it constituted with the latter a semi-solid cake, which moulded itself to the shape of subjacent parts, and effectually prevented hemorrhage. It was particularly in fungous tumours disposed to this accident that M. Rivaillé had found nitric acid useful as a local application. M. Rivaillé also in many instances employed alum for the purpose of arresting the progress of hospital gangrene.

*Spontaneous Dislocation of the Knee.*—M. Palasciano forwarded a communication, in which he endeavoured to establish—first, that the muscle known as "tensor vaginæ femoris" did not deserve that name, but, being inserted by long tendinous fibres to the external condyle of the tibia, its use was to rotate the leg outwards, and to abduct the knee when it is bent. Starting from these anatomical data, M. Palasciano observed that the spontaneous dislocation of the knee was a complicated affection, constituted by the flexion, rotation, and abduction of the knee; displacement of the tibia backwards, of the patella outwards, and often with more or less complete ankylosis. In order to cure this disease, which had hitherto been erroneously considered as irremediable, M. Palasciano proposed the section of the flexor tendons, of that of the rotator externus (tensor v. f.,) of the rectus femoris, and vastus externus, also the division of the external lateral ligaments of the joint: preliminary operations which would permit the rupture of the ankylosis and the surgical reduction of the limb to its natural direction.—*Medical Times.*

